# Microbial 5′-nucleotidases: their characteristics, roles in cellular metabolism, and possible practical applications

**DOI:** 10.1007/s00253-021-11547-w

**Published:** 2021-09-27

**Authors:** Natalia P. Zakataeva

**Affiliations:** Ajinomoto-Genetika Research Institute, 1st Dorozhny Proezd, b.1-1, Moscow, 117545 Russia

**Keywords:** 5′-Nucleotidases (EC 3.1.3.5), Hydrolytic dephosphorylation, 5′-Nucleotidase/UDP-glucose hydrolase, UshA, HADSF phosphatases

## Abstract

5′-Nucleotidases (EC 3.1.3.5) are enzymes that catalyze the hydrolytic dephosphorylation of 5′-ribonucleotides and 5′-deoxyribonucleotides to their respective nucleosides and phosphate. Most 5′-nucleotidases have broad substrate specificity and are multifunctional enzymes capable of cleaving phosphorus from not only mononucleotide phosphate molecules but also a variety of other phosphorylated metabolites. 5′-Nucleotidases are widely distributed throughout all kingdoms of life and found in different cellular locations. The well-studied vertebrate 5′-nucleotidases play an important role in cellular metabolism. These enzymes are involved in purine and pyrimidine salvage pathways, nucleic acid repair, cell-to-cell communication, signal transduction, control of the ribo- and deoxyribonucleotide pools, etc. Although the first evidence of microbial 5′-nucleotidases was obtained almost 60 years ago, active studies of genetic control and the functions of microbial 5′-nucleotidases started relatively recently. The present review summarizes the current knowledge about microbial 5′-nucleotidases with a focus on their diversity, cellular localizations, molecular structures, mechanisms of catalysis, physiological roles, and activity regulation and approaches to identify new 5′-nucleotidases. The possible applications of these enzymes in biotechnology are also discussed.

**Key points**

• *Microbial 5′-nucleotidases differ in molecular structure, hydrolytic mechanism, and cellular localization*.

• *5′-Nucleotidases play important and multifaceted roles in microbial cells*.

• *Microbial 5′-nucleotidases have wide range of practical applications.*

**Graphical abstract:**

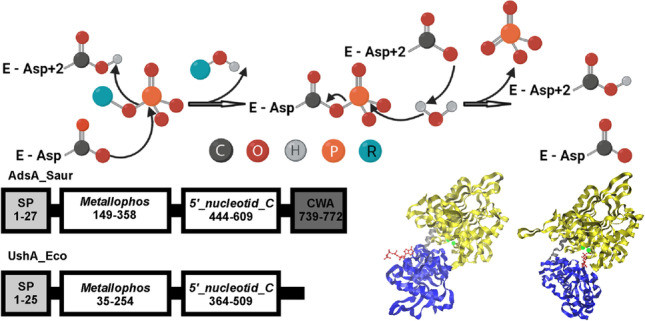

## Introduction

5′-Nucleotidases (EC 3.1.3.5) are enzymes that catalyze the hydrolytic dephosphorylation of 5′-ribonucleotides and 5′-deoxyribonucleotides to their corresponding nucleosides plus phosphate [5′-(deoxy)ribonucleotide + H_2_O <  =  > (deoxy)ribonucleoside + phosphate]. All 5′-nucleotidases characterized to date have relatively broad substrate specificities but show a preference for certain substrates. Many characterized 5′-nucleotidases are multifunctional enzymes that can cleave both phosphate ester and phosphate anhydride bonds and, along with 5′-(deoxy)ribonucleotides, hydrolyze a variety of phosphorylated metabolites, such as phosphoribosyl pyrophosphate, sugar phosphates, complex nucleotides (nicotinamide mononucleotide, nicotinic acid mononucleotide, nicotinamide adenine dinucleotide, flavin adenine dinucleotide), nucleoside diphosphate sugars (for example, UDP-glucose), and CDP-alcohols.

5′-Nucleotidases are widespread among all kingdoms of life and found in different cellular locations (Zimmermann 1992). The well-studied mammalian 5′-nucleotidases have been shown to exist in a membrane-bound, surface-located form or a soluble, cytosolic form (Bianchi and Spychala 2003). The presence of at least seven genes encoding 5′-nucleotidases in the human genome suggests that these enzymes perform important physiological functions. Depending on their cellular location, mammalian 5′-nucleotidases are involved in purine and pyrimidine salvage pathways, nucleic acid repair, cell-to-cell communication, signal transduction, etc. Soluble, cytosolic 5′-nucleotidases, together with nucleoside kinases, control the ribo- and deoxyribonucleotide pools (Hunsucker et al. 2005). Surface-located ecto-5′-nucleotidases are glycosyl phosphatidylinositol (GPI)–linked, membrane-bound glycoproteins that mainly convert extracellular AMP to adenosine. Through binding specific purinergic P1-type receptors, adenosine mediates neurotransmission, conduction, secretion, vasodilation, proliferation, and cell death and inhibits the immune and inflammatory responses and lipolysis (Borowiec et al. 2006). Thus, by influencing the level of proinflammatory extracellular ATP and anti-inflammatory extracellular adenosine, ecto-5′-nucleotidases are involved in cell–matrix or cell–cell interactions and transmembrane signaling and play important roles in immune and inflammatory responses (Zimmermann et al. 2012).

Although the first evidence of microbial nucleotidases was obtained quite a long time ago (Kohn and Reis 1963), active studies of genetic control and the functions of microbial 5′-nucleotidases started relatively recently. To date, microbial 5′-nucleotidases have not been studied as extensively as mammalian 5′-nucleotidases.

The present review summarizes the current knowledge about microbial 5′-nucleotidases, with a focus on their diversity, cellular localizations, molecular structures, mechanisms of catalysis, activity regulation, and physiological roles; approaches to identify these enzymes; and their possible applications in biotechnology.

## Classification of microbial 5′-nucleotidases, their molecular structures, and preferable substrates

All 5′-nucleotidases hydrolyze biologically important phosphate ester linkages, and many have been shown to hydrolyze phosphate anhydride linkages. 5′-Nucleotidases can be classified based on their mechanism of hydrolysis and the type of molecule used as the initial acceptor of the substrate phosphoryl group: an activated water molecule or a nucleophilic amino acid residue (Schultz-Heienbrok et al. 2005). In this review, the catalytic mechanism in which a water molecule is utilized as the initial acceptor of the phosphoryl group is designated the type I catalytic mechanism, while the catalytic mechanism, proceeding in two steps, in which the nucleophilic amino acid residue of the enzyme acts as an intermediate acceptor of the phosphoryl group is designated the type II catalytic mechanism. In microbial cells, these hydrolytic mechanisms are utilized by enzymes present in all possible forms (soluble or surface-located, cell wall–anchored, or membrane-bound) and at all locations (intracellular, periplasmic or extracellular).

Depending on their molecular structure, type of hydrolytic mechanism, and cellular location, microbial 5′-nucleotidases hydrolyze a certain substrate range, exhibit a specific pattern of activity regulation, and play a certain physiological role.

### Secreted 5′-nucleotidases that use the type I catalytic mechanism

**Fig. 1:**
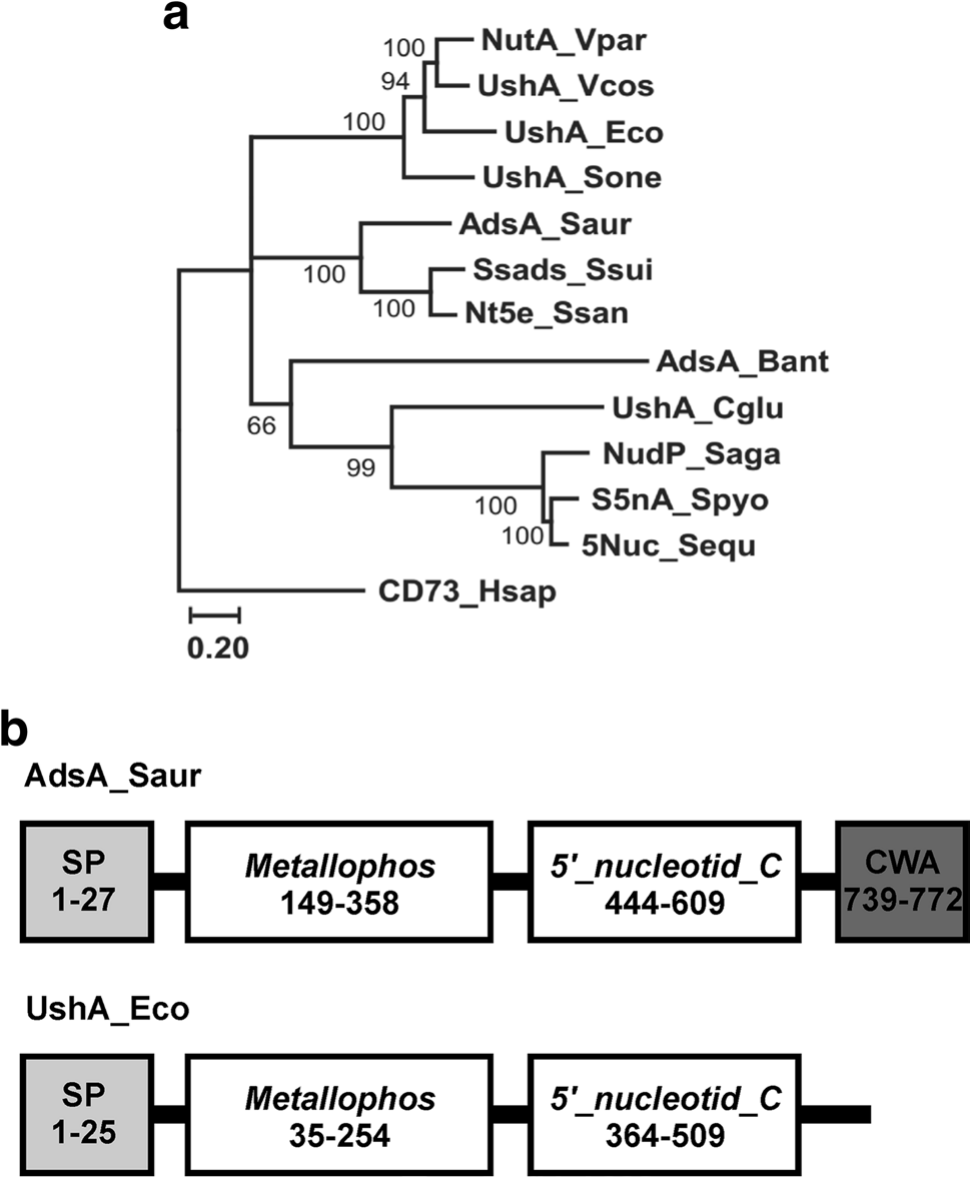
Phylogenetic tree (**a**) and schematic presentation (**b**) of several surface-located and periplasmic 5′-nucleotidases that use the type I catalytic mechanism. CD73_Hsap, *Homo sapiens* CD73 (P21589); UshA_Eco, *E. coli* UshA (P07024); NutA_Vpar, *Vibrio parahaemolyticus* NutA (P22848); UshA_Cglu, *Corynebacterium glutamicum* UshA (WP_011896359); NudP_Saga, *Streptococcus agalactiae* NudP (CDN66659); S5nA_Spyo, *Streptococcus pyogenes* S5nA (Q9A0A2); AdsA_Saur, *Staphylococcus aureus* AdsA (WP_061821283); Ssads_Ssui, *Streptococcus suis* Ssads (CAR45827); Nt5e_Ssan, *Streptococcus sanguinis* Nt5e (AFK32764); 5Nuc_Sequ, *Streptococcus equi* subsp. *zooepidemicus* 5Nuc (AEJ25391); UshA_Vcos, *Vibrio* (*Salinivibrio*) *costicola* UshA (WP_102505627); UshA_Sone, *Shewanella oneidensis* UshA (Q8EFH1); and AdsA_Bant, *Bacillus anthracis* AdsA ( Q6HTQ7). **a** Phylogenetic tree was constructed using maximum likelihood (ML) method. ML analysis was performed with Clustal Omega-generated (https://www.ebi.ac.uk/Tools/msa/clustalo/) multiple sequence alignment using MEGA7 package (Kumar et al. 2016) with 200 bootstrap replicates. WGA + G5 + I model (Whelan and Goldman 2001) was selected as the best-fit model based on both AIC and BIC. The bootstrap values are shown at branching points. **b** Schematic diagram of cell wall–anchored *S. aureus* SdsA (UshA) and periplasmic *E. coli* UshA based on predictions with InterProScan software at EMBL-EBI. White boxes highlight the two typical domains of 5′-nucleotidases of this type: the calcineurin-like phosphoesterase domain (*Metallophos*; PF00149) and 5′-nucleotidase C-terminal domain (*5*_*nucleotid*_*C*; PF02872). Gray and dark gray boxes represent the signal peptide (SP) and cell wall–anchoring domain (CWA), respectively. Amino acid residue numbers are shown in brackets

Despite differences in their localization and their attachment to the cell surface or presence in a soluble form, all secreted (surface-located, periplasmic and extracellular) microbial 5′-nucleotidases that utilize the type I catalytic mechanism characterized to date show low but significant sequence identity with the mammalian ecto-5′-nucleotidase CD73 (Zimmermann 1992; Colgan et al 2006), indicating their common ancestry and similar structures (Volknandt et al. 1991; Sträter 2006) (Fig. 1a).

**Fig. 2:**
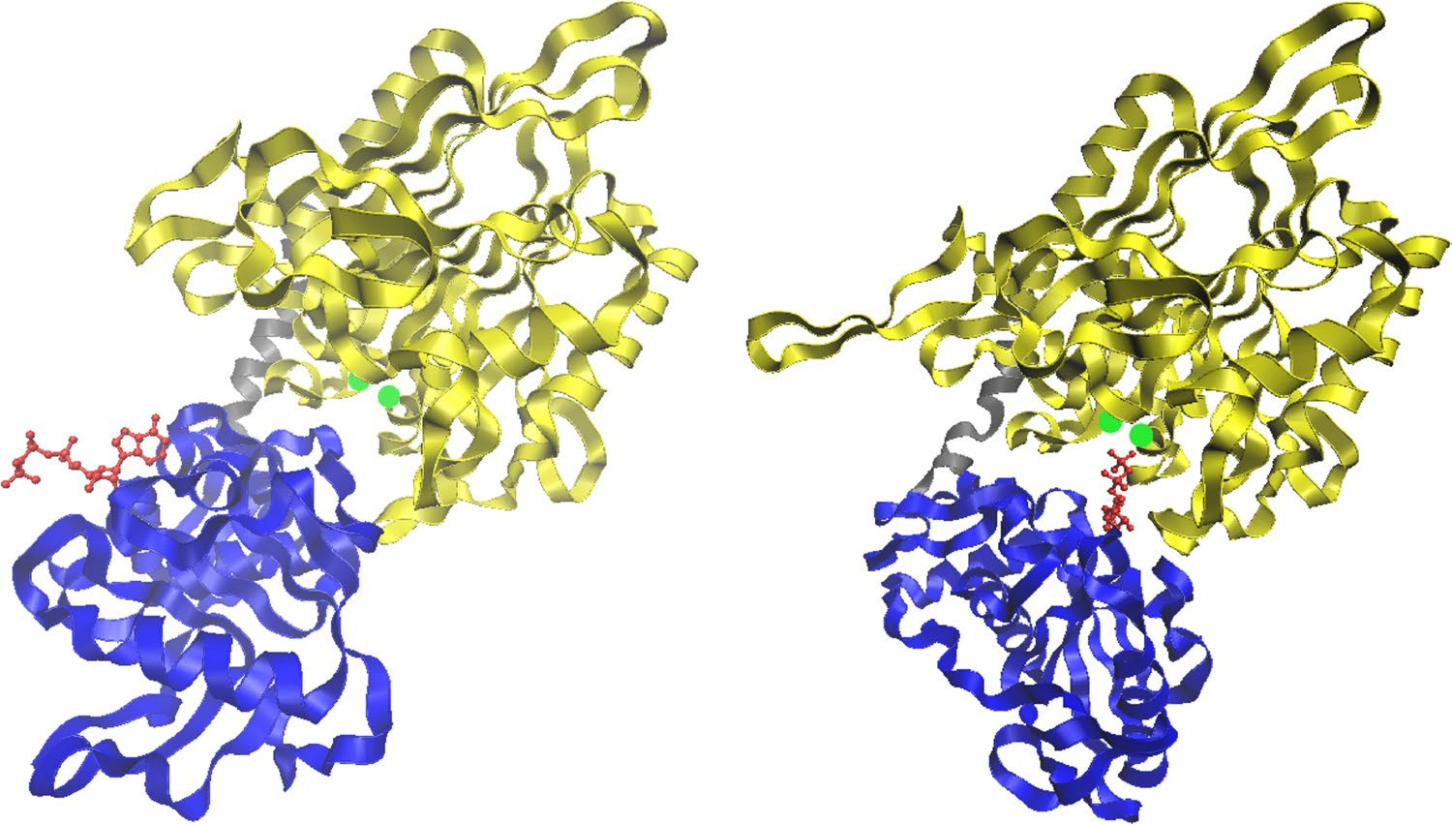
Crystal structure of *E. coli* UshA. Open form of UshA in complex with ATP (PDB: 1HP1) (left) and closed form of UshA with an inhibitor (α,β-methylene-ADP) bound to the active site (PDB: 1HPU, chain C) (right) are shown. The N‐terminal domain (residues 26–342) is shown in yellow, the helix linker (residues 343–362) in gray, the C‐terminal domain (residues 363–550) in blue and two Mn^2+^ ions in green. The substrate and inhibitor are shown in red. ATP and the inhibitor are bound to the same binding site in the C-terminal domain in the open form and closed form, respectively. Upon 96° rotation of the C-terminal domain, the substrate is brought into the proximity of the dimetal center

The catalytic efficiency of 5′-nucleotidases of this type is determined by a combination of two evolutionarily independent domains from which the proteins are formed. The N-terminal domain, which contains a dimetal center and the catalytic dyad Asp-His in the active site, is characteristic of the calcineurin-like phosphoesterase superfamily (PF00149.28) of phosphatases, while the C-terminal domain is characteristic of the 5′-NT superfamily (PF02872.18) (Fig. 1b). These two domains (the metallophosphoesterase and 5′-nucleotidase domains) are linked by an α-helix. The molecular structures and catalytic mechanisms of enzymes of this type were thoroughly investigated with *Escherichia coli* UshA used as a representative (Knofel and Strater 2001; Krug et al. 2013). The C-terminal domain is responsible for binding the substrate base, while the N-terminal domain is responsible for the core catalytic steps. These steps include nucleophilic attack on the phosphate group by a water molecule bound to one of the metal ions, subsequent hydrolysis of the phosphate bond, formation of a phosphoenzyme intermediate, and, finally, the release of orthophosphate from the reaction system. The substrate phosphate group is positioned by hydrogen bonds from amino acids in both the N-terminal and C-terminal domains and by hydrogen bonds from the two metal ions. A 96° domain rotation between the open (inactive) and closed (active) enzyme forms is necessary for enzyme catalytic activity (Fig. 2) (Knofel and Strater 2001; Schultz-Heienbrok et al. 2005). The characterized secreted 5′-nucleotidases that use the type I catalytic mechanism are widely distributed in various bacteria and exist in a membrane-bound/cell wall–anchored, surface-located form and an extracellular or periplasmic soluble form.

#### Surface-located 5′-nucleotidases that utilize the type I catalytic mechanism

Several cell wall–anchored and membrane-bound 5′-nucleotidases that utilize the type I catalytic mechanism have been found and characterized in gram-positive (*Staphylococcus*, *Streptococcus*, and *Bacillus* species) and gram-negative (*Vibrio*, *Shewanella*, and *Legionella* species) bacteria, respectively (Table 1, Fig. 1). Many of these proteins from gram-positive bacteria harbor C-terminal sortase A–specific sorting signals (LPXTG), indicating that their mature products are localized in the cell wall envelope (Thammavongsa et al. 2009). However, the mechanism by which 5′-nucleotidases attach to the cell surface of gram-negative bacteria has not yet been investigated in detail. Lipoproteins are targeted for export across the cytoplasmic membrane by a signal peptide (SP), which is then cut off at the cleavage site, after which the acyl chain attaches to the now accessible amino group of the cysteine residue, completing construction of the membrane anchor (Zückert 2014). A possible SP consisting of the sequence Ser-Leu-Ala-Gly-Cys found at the N-terminus of membrane-bound 5′-nucleotidase from *Vibrio parahaemolyticus* is similar to the consensus signal lipoprotein-cleavage site in gram-negative bacteria (Leu-Leu-Ala-Gly-Cys), suggesting that cell wall anchoring occurs through the mechanism described for lipoproteins.

**Table 1:** Microbial enzymes with 5'-nucleotidase activity

Cellular location	Protein family	The catalytic mechanism/initial phosphoryl acceptor	Source	Protein (Accession number)	Substrates	Metal ion requirement	References
Membrane-bound / cell wall-anchored, surface-located	N-terminal calcineurin-like phosphoesterase domain (*Metallophos*; PF00149) and C-terminal 5´-nucleotidase (*5'_nucleotid_C*; PF02872) domain	Type I / a water molecule	*Staphylococcus aureus* (G +)	AdsA (UshA) (WP_061821283)	AMP, ADP, ATP, dAMP, GDP, GTP	Mg^2+^, Mn^2+^	Thammavongsa et al. 2011
			*Streptococcus sanguinis* (G +)	Nt5e (AFK32764)	AMP, ADP, ATP	Mg^2+^, Ca^2+^	Fan et al. 2012
			*Streptococcus agalactiae* (G +)	NudP (CDN66659)	(d)NMP, (d)NDP, but not (d)NTP	Mn^2+^	Firon et al. 2014
			*Streptococcus equi* subsp. z*ooepidemicus* (G +)	5Nuc (AEJ25391)	AMP, ADP, ATP, dAMP	Mg^2+^, Ca^2+^	Ma et al. 2016
			*Streptococcus pyogenes* (G +)	S5nA (Q9A0A2)	AMP, ADP, dAMP, CMP, GMP, but not ATP	Mg^2+^, Mn^2+^, Ca^2+^	Zheng et al. 2015
			*Streptococcus suis* (G +)	Ssads (CAR45827)	AMP, ADP, ATP	Mn^2+^	Liu et al. 2014
			*Streptococcus iniae* (G +)	S5nAi (WP_003099850)	ADP, AMP, dAMP, GMP, CMP, TMP, but not ATP	Mg^2+^, Mn^2+^, Ca^2+^	Soh et al. 2018
			*Bacillus anthracis* (G +)	AdsA **(**Q6HTQ7)	dAMP	Mn^2+^	Thammavongsa et al. 2009
			*Vibrio parahaemolyticus* (G −)	NutA (UshA) (P22848)	AMP, ADP, ATP	Cl^−^, Mg^2+^, Mn^2+^, Co^2+^	Itami et al. 1989; Sakai et al. 1987
			*Vibrio* (*Salinivibrio*) *costicola* (G −)	UshA (WP_102505627)	NMP, NDP, NTP	Cl-	Bengis-Garber and Kushner 1981
			*Shewanella violacea* (G −)	UshA (WP_041419915)	AMP, ATP, GTP	Mg^2+^, Mn^2+^	Kuribayashi et al. 2017
			*Shewanella amazonensis* (G −)	UshA (WP_011760134)			
	Class C acid phosphatase (cd07534); belong to HAD-like superfamily (IPR036412)	Type II / nucleophilic amino acid residue	*Helicobacter pylori* (G −)	HppA (Q6UC93)	NMP	Cu^2+^, Ni^2+^, Co^2+^, Mg^2+^	Reilly and Calcutt 2004
			*Clostridium perfringens* (G +)	CppA (ACB11490)	UMP, GMP, AMP, 3′ TMP, ATP, 3′ AMP, FMN, R5P, pyridoxal phosphate, ADP, 2′ AMP, NADP	Cu^2+^, Co^2+^, Cr^2+^	Reilly et al. 2009
			*Chryseobacterium meningosepticum* (*Elizabethkingia meningoseptica*) (G −)	OlpA (O08351)	NMP, 3′ AMP	Cu^2+^, Mg^2+^	Passariello et al. 2003
			*Haemophilus influenzae* (G −)	Lipoprotein Hel [e (P4)] (WP_118891437)	Aryl phosphates	Cu^2+^	Reilly et al. 1999
	Phosphoesterase family (PF04185), consists of two domains: alkaline-phosphatase-like, core domain superfamily (IPR017850) and Phosphoesterase (IPR007312)	Type II / nucleophilic amino acid residue	*Francisella tularensis* (G −)	AcpA (WP_003027314)	NMP, NTP, NDP, FMN, NMN, NADP etc	Me^2+^	Mohapatra et al. 2007; Reilly et al. 2006
Periplasmic	N-terminal calcineurin-like phosphoesterase domain (*Metallophos*; PF00149) and C-terminal 5´-nucleotidase (*5'_nucleotid_C*; PF02872) domain	Type I / a water molecule	*Escherichia coli* (G −)	UshA (P07024)	(d)NTP, bis(5'-nucleosidyl)polyphosphates, UDP sugars, CDP-alcohols, NAD(H)	Zn^2+^, Co^2+^or Mg^2+^, depending on type of hydrolase activity	Alves-Pereira et al. 2008; Ruiz 1989; Glaser et al. 1967; Neu 1967; Wang et al. 2014
			*Vibrio cholerae* (G −)	UshA (Q9KQ30)	dNTP	-	McDonough et al. 2016
			*Yersinia intermedia* (G −)	UshA (A4URQ8)	(d)NTP, bis(5'-nucleosidyl)polyphosphates, UDP sugars, CDP-alcohols	Co^2+^ or Mg^2+^, depending on the type of hydrolase activity	Alves-Pereira et al. 2008; Neu 1967
			*Shewanella oneidensis* (G −)	UshA (Q8EFH1)	FAD, AMP	-	Covington et al. 2010
			*Haemophilus influenzae* (G −)	NadN (P44569)	AMP, NAD + , UDP sugars	Zn2 +	Garavaglia et al. 2011
			*Klebsiella aerogenes* (*Enterobacter aerogenes*) (G −)	UshA (A0A0H3FPS2)	UDP sugars	-	Lee et al. 2000
	Class A acid phosphatase (IPR001011)	Type II / nucleophilic amino acid residue	*Morganella morganii* (*Proteus morganii*) (G −)	PhoC (P28581)	UMP, AMP, 3’-UMP, 3’-AMP	-	Thaller et al. 1994
	Acid phosphatase class B-like (IPR005519) or HAD superfamily, subfamily IIIB (acid phosphatase) (PF03767)		*Morganella morganii* (*Proteus morganii*) (G −)	NapA (AphA) (Q59544)	NMP, 3’ NMP, aryl phosphates, β-glycerophosphate, sugar phosphates, but not on diesters	Mg^2+^, Co^2+^, Zn^2+^	Thaller et al. 1995
			*Salmonella enterica* (G −)	AphA (QQL85243)	UMP, 3' -UMP, pNPP and a-naphthyl phosphate, but not diesters	Mg^2+^, Co^2+^, Zn^2+^	Uerkvitz and Beck 1981
			*Escherichia coli* (G −)	AphA (P0AE22)	3'-(d)NMP, (d)NMP	Mg^2+^	Thaller et al. 1997
	Alkaline phosphatase (PF00245)		*Escherichia coli* (G −)	PhoA (P00634)	Wide variety of phosphate monoesters, including 5´-ribo- and 5´-deoxyribonucleotides	Mg^2+^, Zn^2+^	Garen and Levinthal 1960
Extracellular	N-terminal calcineurin-like phosphoesterase domain (*Metallophos*; PF00149) and C-terminal 5´-nucleotidase (*5'_nucleotid_C*; PF02872) domain	Type I / a water molecule	*Corynebacterium glutamicum* (G +)	UshA (WP_011896359)	GMP, IMP, XMP, AMP, UMP, CMP, ADP, ATP, dATP	Co^2+^, Ca^2+^, Mg^2+^, depending on the type of hydrolase activity	Rittmann et al. 2005
	PRK09419 Superfamily (multifunctional 2',3'-cyclic-nucleotide 2'-phosphodiesterase/3'-nucleotidase/5'-nucleotidase), contains N-terminal calcineurin-like phosphoesterase domain (*Metallophos*; PF00149) and C-terminal 5´-nucleotidase (*5'_nucleotid_C*; PF02872) domain		*Bacillus subtilis* (G +)	YfkN (BAA23404.1)	3´-nucleotides, 2′3'-cyclic-nucleotides and 5'-nucleotides	-	Chambert et al. 2003
Intracellular	HAD-superfamily hydrolase, subfamily IIA (IPR006357)	Type II / nucleophilic amino acid residue	*Escherichia coli* (G −)	YigB (P0ADP0)	5-amino-6-(5-phospho-D-ribitylamino)uracil, FMN	Mg^2+^	Haase et al. 2013; Kuznetsova et al. 2006
			*Bacillus subtilis* (G +)	YutF (NucF) (WP_003243196)	R5P, XMP, PRPP, IMP, GMP, dGMP, dIMP	Mg^2+^	Zakataeva et al. 2016
			*Escherichia coli* (G −)	UmpH (NagD) (C3TJ42)	UMP, GMP, (d)NTP, G1P	Mg^2+^	Kuznetsova et al. 2006; Tremblay et al. 2006
	HAD-superfamily hydrolase, subfamily IA (IPR006439)		*Escherichia coli* (G −)	YjjG (P0A8Y1)	dTMP, dUMP, UMP, non-canonical pyrimidine derivatives (5-fluoro-2′-deoxyuridine, 5-fluorouridine, 5-fluoroorotic acid, 5-fluorouracil etc.)	Mn^2+^, Mg^2+^	Kuznetsova et al., 2006; Proudfoot et al. 2004; Titz et al. 2007
			*Bacillus subtilis* (G +)	YitU (P70947)	FMN, ARPP, (d)NMP (dAMP, GMP, dGMP, CMP, AMP, XMP, IMP), AICAR-P	Mg^2+^	Sarge et al. 2015; Yusupova et al. 2020
	HAD-superfamily hydrolase, subfamily IIB (IPR006379)		*Bacillus subtilis* (G +)	YcsE (P42962)	ARPP, FMN, IMP, AMP, GMP, CMP, UMP, G6P	Mg^2+^	Sarge et al. 2015; Terakawa 2016
	HAD-superfamily hydrolase, subfamily IG, 5'-nucleotidase (IPR008380)		*Legionella pneumophila* (G −)	cN-II (Q5ZZB6)	GMP, dGMP, IMP, pNPP	Mg^2+^	Srinivasan et al. 2014
	HD-domain phosphohydrolases (PF01966)	Type I / a water molecule	*Escherichia coli* (G −)	YfbR (P76491)	dNMP	Co^2+^, Mn^2+^, Cu^2+^	Proudfoot et al. 2004; Zimmerman et al. 2008
	SurE family (PF01975) or survival protein SurE (IPR030048)	Type I / a water molecule	*Escherichia coli* (G −)	UmpG (SurE) (P0A840)	(d) NMP, 3′-AMP, polyphosphates with the preference for short-chain-length substrates (P_20–25_)	Mn^2+^, Co^2+^, Ni^2+^, Mg^2+^	Proudfoot et al. 2004

The abbreviations used are *NMN*, nicotinamide mononucleotide; *FMN*, flavin mononucleotide, *NADP*, nicotinamide adenine dinucleotide phosphate; *FAD*, flavin adenine dinucleotide; *AICAR-P*, 5-aminoimidazole-4-carboxamide-1-β-D-ribofuranosyl 5′-monophosphate; *ARPP*, 5-amino-6-ribitylamino-2,4(1H,3H)-pyrimidinedione 5'-phosphate; *G1P*, glucose 1-phosphate; *G6P*, glucose 6-phosphate; *R5P*, ribose 5-phosphate; *PRPP*, 5-phosphoribosyl 1-pyrophosphate*; pNPP*, p-nitrophenyl phosphate; *(d)NMP/(d)NDP/(d)NTP*, ribonucleoside or deoxyribonucleoside mono-, di- and triphosphates. The accession numbers correspond to the UniProt or NCBI databases; (G −) and (G +)—gram-negative and gram-positive bacteria; –—not found

Despite differences in their preferred substrates, pH ranges, and metal ion requirements, the cell wall–anchored and membrane-bound 5′-nucleotidases that exploit the type I catalytic mechanism characterized to date show two activities, 5′-nucleotidase activity to hydrolyze nucleoside monophosphates and triphosphate diphosphohydrolase activity to hydrolyze nucleoside tri- and diphosphates (Bengis-Garber and Kushner, 1981; Tamao et al. 1991; Thammavongsa et al. 2009; Thammavongsa et al. 2011; Fan et al. 2012; Firon et al. 2014; Liu et al. 2014; Zheng et al. 2015; Ma et al. 2017; Soh et al. 2018). Most 5′-nucleotidases of this type predominantly hydrolyze adenylate nucleotides, AMP, ADP, ATP, and, to a lesser extent, guanylate nucleotides. Some of the enzymes of this type can use dAMP or dGMP as a substrate to generate deoxyadenosine and deoxyguanosine, respectively (Thammavongsa et al. 2013; Srinivasan et al. 2014; Zheng et al. 2015) (Table 1).

#### Periplasmic and extracellular 5′-nucleotidases that utilize the type I catalytic mechanism

Soluble, periplasmic, microbial 5′-nucleotidases exhibit rather high levels of amino acid sequence identity with surface-located, vertebrate, and microbial 5′-nucleotidases that utilize the type I catalytic mechanism. These proteins are expressed as immature precursors, which, upon export to the periplasm, undergo maturation by proteolytic cleavage of a signal sequence that targets the precursor protein to the periplasm (Burns and Beacham 1986).

The well-studied periplasmic, microbial 5′-nucleotidase UshA from *E. coli* and orthologous proteins from other bacteria belong to a large, widely distributed, multifunctional family of 5′-nucleotidase/UDP-glucose hydrolase proteins.

The periplasmic 5′-nucleotidases in this family cleave both terminal phosphate ester and phosphate anhydride bonds and possess extremely broad substrate specificity (Table 1), combining 5′-nucleotidase and CDP-alcohol/UDP-sugar/dinucleoside polyphosphate hydrolase activities. All 5′-ribonucleotides and 5′-deoxyribonucleotides (Neu 1967), CDP-alcohols (Alves-Pereira et al. 2008), UDP-sugar pyrophosphates (Glaser et al. 1967), bis(5′-nucleosidyl)polyphosphates (Ruiz et al. 1989), NAD(H) (Wang et al. 2014), and the nonnatural substrates bis(p-nitrophenyl)phosphate and p-nitrophenyl phosphate (Neu 1967) were shown to be substrates of *E. coli* UshA. UshA is a highly efficient periplasmic phosphohydrolase that hydrolyzes 5′-AMP and CDP-alcohols with *K*_m_ values at the µmolar level and very high *k*_cat_/*K*_m_ values of approximately 10^8^ M^−1^ s^−1^ for its preferred substrates (Alves-Pereira et al. 2008).

Proteins orthologous to UshA have been found in many gram-negative bacteria (*Yersinia intermedia*, *Vibrio* *cholerae*, *Haemophilus influenzae*, *Shewanella oneidensis*, etc.) (Alves-Pereira et al. 2008; Covington et al. 2010; Garavaglia et al. 2012; McDonough et al. 2016) and in the gram-positive bacteria *Corynebacterium glutamicum* (Rittmann, et al. 2005) and *Bacillus subtilis* (Chambert et al. 2003) (Table 1).

Contrary to *E. coli* UshA, which exhibits very low activity against FAD, its ortholog from the metal-reducing Gammaproteobacterium *S. oneidensis*, which shares approximately 50% amino acid identity with *E. coli* UshA, can hydrolyze FAD in the periplasmic space. The products of FAD hydrolysis are FMN, which diffuses through outer membrane porins, and AMP, which is also dephosphorylated by UshA and reassimilated by the cell (Covington et al. 2010). The UshA ortholog from *Enterobacter aerogenes* possesses confirmed UDP-sugar hydrolase activity (Lee et al. 2000)*.*

Soluble, extracellular 5′-nucleotidases have been isolated from the culture medium of some gram-negative bacteria, such as *V. cholerae* (Punj et al. 2000), *Burkholderia cepacia* (Melnikov at al. 2000), and *Pseudomonas aeruginosa* (Zaborina et al. 1999). However, gene expression and fractionation experiments have not been reported by these authors; therefore, the localizations of these enzymes have not been clearly established.

The localization of the UshA ortholog from the gram-positive bacterium *C. glutamicum* is also uncertain. Although *C. glutamicum* UshA was shown to be present in a soluble, extracellular form, the amino acid sequence of this protein may contain a C-terminal transmembrane helix (Rittmann et al. 2005), indicating the possibility of membrane attachment under certain conditions. This phenomenon has also been described for some vertebrate ecto-5′-nucleotidases, which were shown to exist in two forms, membrane-bound and soluble, extracellular forms (Zimmermann et al. 2012). *C. glutamicum* UshA has demonstrated activity against guanosine monophosphate (GMP), inosine monophosphate (IMP), AMP, CMP, UMP, ADP, ATP, dATP, and UDP-glucose (Rittmann et al. 2005). YfkN, another homolog of UshA secreted into the extracellular space by the gram-positive bacterium *B. subtilis*, is encoded by the gene produced from natural fusion of the *cpdB* (3′-nucleotidase/2′3′ cyclic nucleotidase) and *ushA* (5′-nucleotidase) genes. The translated 143.5-kDa protein is the largest among all proteins expressed by *B. subtilis*. This trifunctional nucleotide phosphoesterase possesses extremely broad substrate specificity towards 3′-nucleotides, 2′3′-cyclic nucleotides, and 5′-nucleotides, with greater activity against 2′3′-cyclic nucleotides than 5′-nucleotides (Chambert et al. 2003).

### Membrane-bound and periplasmic phosphohydrolases with 5′-nucleotidase activity that use the type II catalytic mechanism

In addition to 5′-nucleotidases, which use a water molecule as an initial phosphoryl group acceptor, bacteria have many secreted, membrane-bound phosphohydrolases and periplasmic, so-called nonspecific phosphohydrolases, which use the type II catalytic mechanism. These enzymes hydrolyze monophosphate esters in two steps through the formation of a phosphoenzyme intermediate, which can transfer its phosphate group to water (dephosphorylation) or another acceptor (transphosphorylation). Catalysis can proceed with or without metal ion cofactors. Nonspecific phosphohydrolases are active against a wide range of structurally unrelated substrates, including 3′- and 5′-nucleoside monophosphates, nucleoside diphosphates, nucleoside triphosphates, hexose and pentose phosphates, and other phosphorylated compounds. These enzymes exhibit optimal catalytic activities under alkaline or acidic conditions and are thus named alkaline phosphatases (EC 3.1.3.1) or acid phosphatases (EC 3.1.3.2), respectively. Based on their subcellular localization and conserved amino acid sequence motifs, acid phosphatases are in turn divided into at least 3 classes: A (IPR001011; https://www.ebi.ac.uk/interpro/entry/InterPro/IPR001011/), B (IPR005519; https://www.ebi.ac.uk/interpro/entry/InterPro/IPR005519/), and C (cd07534; https://www.ebi.ac.uk/interpro/entry/cdd/CD07534/) (Vincent et al. 1992; Rossolini et al. 1998; Gandhi and Chandra 2012).

Membrane-bound, surface-located acid phosphatases and periplasmic acid phosphatases with 5′-nucleotidase activity have been found in both gram-positive and gram-negative bacteria. Some are shown in Table 1. Secreted into the periplasmic space, AphA from both *E. coli* and *Salmonella enterica*, NapA from *Morganella morganii*, and other orthologous proteins from various pathogenic bacteria (Calderone et al. 2006) are class B acid phosphatases (InterPro family 005519) or, since they contain the haloacid dehalogenase (HAD) fold, belong to the HAD superfamily, subfamily IIIB (Acid phosphatase) (PF03767). These enzymes contain two pairs of absolutely conserved aspartic acid residues, which are essential for phosphomonoesterase activity and separated by a linker region (a DDDD motif). The enzymes require Mg^2+^ ions as cofactors and are active against various organic phosphomonoesters, including *p*-nitrophenyl phosphate (*p*NPP) and 5′- and 3′-nucleoside monophosphates, but not diesters (Table 1) (Thaller et al. 1995, 1997; Uerkvitz and Beck 1981). For example, AphA from *E. coli* exhibits optimal activity at a slightly acidic pH and is active against several substrates, including *p*NPP, 5′- and 3′-mononucleotides, β-glycerol phosphate, and sugar phosphates (Thaller et al. 1997). This enzyme exhibits a strong preference for 5′- and 3′-mononucleotide substrates (k_*cat*_/KM usually > 10^6^ M^−1^ s^−1^) and other organic phosphomonoesters with an aromatic ring moiety. AphA can also catalyze the transfer of phosphate from phosphomonoester donors, such as *p*NPP and 3′- or 5′-mononucleotides, to the hydroxyl groups of other acceptors, such as nucleosides.

Class C acid phosphatases are distantly related to class B acid phosphatases and characterized by the presence of a DDDD motif (Thaller et al. 1998). However, class C phosphatases are anchored to the membrane by an N-terminal lipid group (Malke 1998; Rossolini et al. 1998; Reilly et al. 2009). Several orthologous proteins in this class that possess activity against, among other substrates, 5′-nucleoside monophosphates have been found in the pathogenic bacteria *Clostridium perfringens*, *Pasteurella multocida*, *Helicobacter pylori*, *Bacillus anthracis*, *Staphylococcus aureus*, *Streptococcus equisimilis*, *Chryseobacterium meningosepticum*, etc*.* and characterized (Malke 1998; Passariello et al. 2003; Reilly and Calcutt 2004; Felts et al. 2006; Reilly et al. 2009). Thus, HppA from *H. pylori* hydrolyzes *p*NPP and 5′-nucleoside monophosphates in the presence of the divalent cations Cu^2+^, Ni^2+^, Co^2+^, and Mg^2+^. Recombinant HppA has relatively narrow substrate specificity and exhibits the greatest activity against 5′-nucleoside monophosphates (Reilly and Calcutt 2004). The acid phosphatase OlpA from *C. meningosepticum*, which causes neonatal meningitis, is a broad-spectrum nucleotidase that efficiently hydrolyzes nucleotide monophosphates but shows a strong preference for 5′-nucleotides and 3′-AMP. OlpA can also hydrolyze sugar phosphates and β-glycerol phosphate, although with lower efficiency (Passariello et al. 2003). The secreted acid phosphatase AcpA from *Francisella tularensis*, which belongs to the phosphoesterase family, contains an alkaline phosphatase-like core domain and hydrolyzes the nucleotides AMP and ATP among many other phosphorylated compounds (Reilly et al. 1996, 2006).

Another nonspecific phosphohydrolase, the *E. coli* alkaline phosphatase *PhoA*, is a homodimeric, periplasmic enzyme that catalyzes the hydrolysis and transphosphorylation of a wide variety of phosphate monoesters, including 5′-ribonucleotides and probably 5′-deoxyribonucleotides (Garen and Levinthal 1960). The enzymatic reaction of PhoA proceeds through a phosphoserine intermediate (the type II catalytic mechanism), which used three metal ions for catalysis (Stec et al. 2000).

### Intracellular microbial 5′-nucleotidases

#### Intracellular 5′-nucleotidases that use the type II catalytic mechanism

**Fig. 3:**
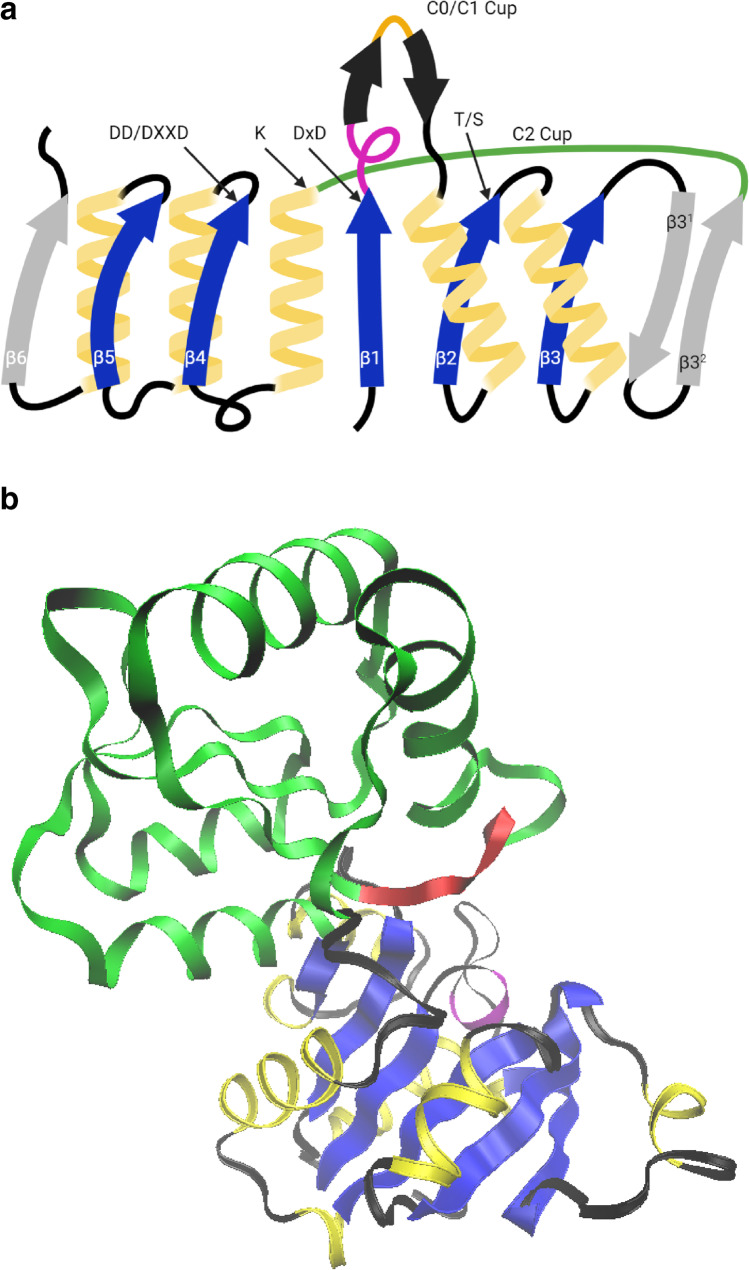
**a** Schematic representation of the classic HADSF Rossmann core domain, which consists of consecutive repeats of a motif composed of a β-strand (blue or gray arrow), a connecting loop (black line), and an α helix (yellow helix). The conserved and nonconserved strands, which are not found in all members, are shown as blue and gray arrows, respectively (the arrow points towards the C-terminal end). The C0/C1 and C2 cap insertion points are shown in orange and green, respectively. Amino acid residues conserved in the Rossmann core domain of all HADSF members are shown. **b** Ribbon diagram representation of the 3D structure of the HADSF subfamily IIA member *E. coli* UmpH (NagD) (PDB: 2C4N). Secondary structure elements are colored in the same colors as in (**a)**, the substrate specificity loop is shown in red

Most intracellular 5′-nucleotidases characterized to date belong to the haloacid dehalogenase superfamily (HADSF). This ubiquitous protein superfamily includes enzymes that catalyze carbon or phosphoryl group-transfer reactions with a diverse range of substrates. All HADSF members contain a highly conserved “core” domain characterized by a three-layered α/β sandwich composed of repeating β–α units (coined the Rossmann-like or Rossmannoid fold) that support a catalytic scaffold and insert, termed the “cap” or “capping” domain, that desolvates the active site for catalysis and provides substrate specificity determinants (Fig. 3a) (Burroughs et al. 2006; Allen and Dunaway-Mariano 2009). Based on the type of cap inserted (C0, C1, or C2 (C2A/C2B)) and their topology and location, HADSF members are divided into four subfamilies: I, IIA, IIB, and III. The HADSF catalytic scaffold is composed of four loops containing the amino acid residues of motifs I–IV. Motif I (DXDX(T/V), where D is aspartate and X is any amino acid) contains two aspartate residues that coordinate the Mg^2+^ cofactor. The first aspartate residue induces nucleophilic attack of the phosphate of the substrate, and the second residue donates a proton to the remaining nucleoside. Motif II (T/S), which is characterized by the presence of a highly conserved threonine or serine residue at its end, and motif III (K/R), which contains a conserved lysine residue, are jointly involved in the stabilization of intermediate products of the hydrolysis reaction. Motif IV contains one of three basic signatures, DD, GDXXXD, or GDXXXXD, and its acidic residues, together with the acidic residues of motif I, facilitate coordination of the Mg^2+^ ion at the active site (Burroughs et al. 2006). The active site is located at the interface between the core and cap domains, with the catalytic residues from the core domain and most of the residues that determine substrate specificity from the cap domain (Fig. 3b).

**Fig. 4:**
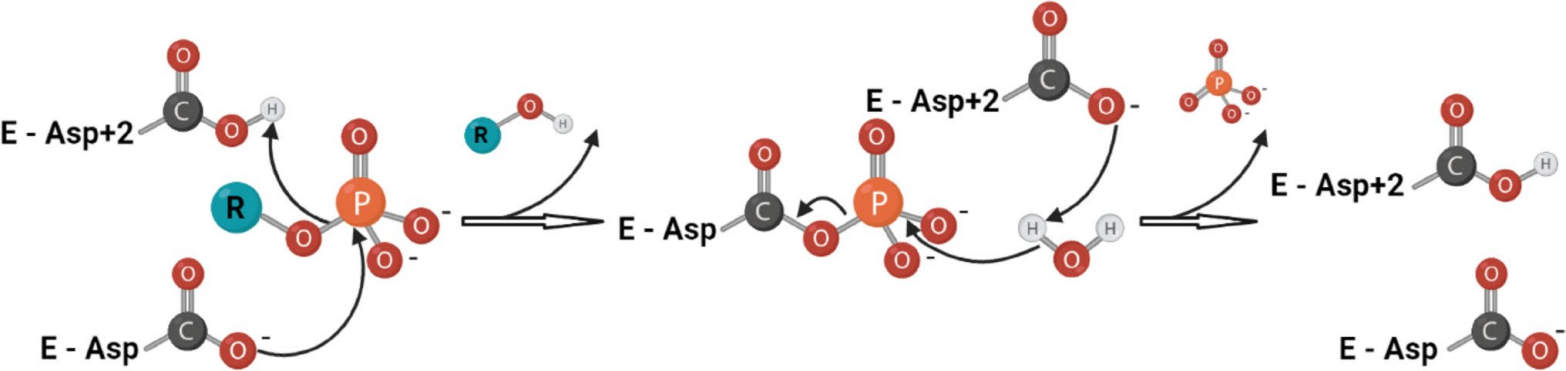
Schematic representation of the general catalytic mechanism of phosphohydrolase members of the HADSF. Catalysis proceeds through an aspartyl-phosphate intermediate

Most HADSF enzymes (approximately 80%) are phosphate monoester hydrolases (phosphatases). These enzymes use the type II catalytic mechanism, catalyzing the dephosphorylation of substrates via a two-step phosphoaspartyltransferase mechanism. The substrate binds with its phosphate and ribose moieties in the core domain and its base moiety in the cap domain. The first step of the enzymatic reaction is nucleophilic attack of the phosphoryl group of the substrate by an aspartate residue in motif I, leading to the formation of a phosphoenzyme intermediate. The second step is hydrolysis of aspartyl-phosphate intermediate and formation of inorganic phosphate (Fig. 4).

Through electrostatic stabilization, the divalent metal cofactor Mg^2+^ assists in positioning of the nucleophile and substrate phosphoryl group to bring them within close proximity and neutralizes the transition state charge.

Microbial genomes can contain from very few to several dozen genes encoding HADSF phosphatases. Multiple genes that encode soluble HADSF phosphatases belonging to various HADSF subfamilies have been identified in the genomes of gram-negative (Proudfoot et al. 2004; Kuznetsova et al, 2006) and gram-positive (Sarge et al. 2015; Terakawa et al. 2016; Zakataeva et al. 2016; Yusupova et al. 2020) bacteria, and their substrate specificities and the biochemical properties of their products have been studied. An important feature of these enzymes is that, unlike the classical “one enzyme for one substrate” model, most HADSF phosphatases possess remarkably broad and overlapping but nonidentical substrate profiles. In general, intracellular HADSF nucleotidases/phosphatases exhibit lower catalytic efficiency than membrane-bound, cell wall–anchored, or extracellular nucleotidases. Thus, it might be necessary to prevent intracellular nucleotide pool depletion. Along with deoxyribo- and ribonucleoside tri-, di-, and monophosphates, HADSF phosphatases can hydrolyze intermediates of various metabolic pathways, sugar phosphates and polyphosphates. Remarkably, most of these enzymes can also recognize small phosphodonors (for example, acetyl phosphate, carbamoyl phosphate, and phosphoramidate), which are usually used for autophosphorylation of the receiver domains of two-component signal transduction systems (Kuznetsova et al. 2006).

The well-studied *E. coli* UMP phosphatase UmpH (NagD) can hydrolyze deoxyribo- and ribonucleoside tri-, di-, and monophosphates, as well as polyphosphate and glucose-1-P (Kuznetsova et al. 2006) but demonstrates the greatest specificity for UMP and GMP (Tremblay et al. 2006; Reaves et al. 2013). Its ortholog from *B. subtilis* and *Bacillus amyloliquefaciens*, YutF, dephosphorylates various purine and pyrimidine 5′-nucleotides, showing a preference for 5′-nucleoside monophosphates and, specifically, 5′-XMP. Recombinant YutF also exhibits phosphohydrolase activity against nucleotide precursors, ribose-5-phosphate and 5-phosphoribosyl-1-pyrophosphate (Zakataeva et al. 2016). The other *B. subtilis* HADSF phosphatases YcsE, YwtE, and YitU were shown to be involved in riboflavin (RF) biosynthesis, catalyzing the dephosphorylation of 5-amino-6-ribitylamino-2,4(1H,3H)-pyrimidinedione 5′-phosphate (ARPP) in the de novo RF pathway and hydrolyzing FMN to RF (Sarge et al. 2015; Yusupova et al. 2020). Similar to all HADSF phosphatases, YcsE and YitU possess broad substrate specificity, demonstrating 5′-nucleotidase activity against various 5′-nucleotides (Terakawa et al. 2016; Yusupova et al. 2020). The other *E. coli* HADSF phosphatases YigB and YbjI preferentially use FMN as a substrate, but the paralogous protein SerB was shown to dephosphorylate phosphoserine (Kuznetsova et al. 2006).

#### Intracellular 5′-nucleotidases that use the type I catalytic mechanism

Several studied intracellular, microbial 5′-nucleotidases that utilize the type I catalytic mechanism are metalloproteins belonging to the histidine–aspartate (HD) domain superfamily (HDDSF). These family members are characterized by a tandem HD dyad that coordinates metal ions. These enzymes act as phosphohydrolases and utilize a two-metal-ion mechanism to catalyze a phosphoryl-to-water molecule transfer reaction. They exhibit phosphomonoesterase and phosphodiesterase activity against a broad range of substrates (Zimmerman et al. 2008; Langton et al. 2020). The representative HDDSF in *E. coli*, 5′-nucleotidase YfbR, is a conserved HD domain phosphohydrolase with more than 100 orthologs in bacteria, archaea, and eukaryotes. YfbR is a dimeric 5′-deoxyribonucleotidase specific to 2′-deoxyribonucleotide-5′-monophosphates (dNMPs) that hydrolyzes dAMP with a kcat/KM of 25.6 × 10^3^ M^−1^ s^−1^ (Zimmerman et al. 2008). Co^2+^ and, to a lesser extent, Mn^2+^, and Cu^2+^ are effective cofactors for YfbR hydrolysis.

Some microbial cells also have intracellular enzymes with 5′-nucleotidase activity that belong to another family, the SurE family (PF01975). The members of this evolutionarily conserved group of proteins, which contain a characteristic N-terminal aspartate-aspartate (DD) motif and possess 5′- and 3′-nucleotidase and exopolyphosphatase activities, are found in eubacteria (except for gram-positive bacteria), archaea, and eukaryotes (Proudfoot et al. 2004). The molecular structures of some members of the SurE family—SurE from *Thermotoga maritima* (Lee et al. 2001; Zhang et al. 2001), *Thermus thermophilus* (Iwasaki and Miki 2007), and *Pyrobaculum aerophilum* (Mura et al. 2003)—have been solved, demonstrating that SurE proteins contain an N-terminal functional domain that resembles a Rossmann fold and extended a C-terminal domain, which is important mainly for maintaining the oligomeric state of the protein. The catalytic mechanism of SurE family members has not been absolutely clarified, but the mechanism is thought to proceed by the type I reaction and use a water molecule as the initial phosphoryl group acceptor. The divalent metal ions required for catalysis are bound in the active center. A strictly conserved asparagine residue (pos. 94 in *T. thermophilus* SurE) is involved in coordinating the metal ion and orientating the water molecule for a nucleophilic attack. Although the optimal metal ion differs between SurE proteins and with different substrates, Mg^2+^ is generally preferred to promote the phosphatase activity of SurE.

## Biological roles of microbial 5′-nucleotidases

Similar to vertebrate 5′-nucleotidases, microbial 5′-nucleotidases have very diverse biological functions that depend on the location of the enzyme in the cell.

### Secreted 5′-nucleotidases

Among bacteria in which surface-located (membrane-bound or cell wall–anchored) 5′-nucleotidases have been found are many pathogenic and conditionally pathogenic species (Table 1). It is well known that surface-exposed proteins often play an important role in the interaction between pathogenic bacteria and their host. Indeed, surface-located 5′-nucleotidases were shown to be clinically relevant virulence factors in pathogenic bacteria. In *S. aureus* and other gram-positive cocci, 5′-nucleotidases of this type increase extracellular concentrations of the potent immunosuppressive molecule adenosine, thereby helping these bacteria compromise the host’s immune defenses and survive in host tissues during infection (Thammavongsa et al. 2009). A similar protective role plays surface-located and extracellular bacterial 5′-nucleotidases, which are specific to dAMP. Together with extracellular nucleases, they convert the DNA from neutrophil extracellular traps (NETs) (which are composed of neutrophil DNA and antimicrobial peptides and allow neutrophils to kill invading pathogens) to deoxyadenosine, which triggers the caspase-3-mediated death of immune cells (Fan et al. 2012; Thammavongsa et al. 2013; Ma et al. 2016).

Although the function of surface-located 5′-nucleotidases from gram-negative pathogenic bacteria as virulence factors has not been directly shown, it could be supposed that these enzymes may also control the immune response of the host by mediating the concentrations of pro- and anti-inflammatory compounds. This assumption is supported by the fact that secreted *V. cholerae* 5′-nucleotidases are involved in suppressing cellular responses during host infection (Punj et al. 2000). Moreover, we can speculate that chronic, prolonged ulceration and the absence of an inflammatory response to infections of the skin and human tissues due to the invasion of *Shewanella* spp. (Sharma and Kalawat 2010) may be associated with the suppression of immune responses by membrane-bound, surface-located 5′-nucleotidases from this bacterium.

Class C acid phosphatases on the surface of intracellular pathogens have also been shown to act as virulence factors that increase intracellular survival through suppression of the respiratory burst (Reilly et al. 1996; Mohapatra et al. 2007).

Membrane-bound, surface-located, bacterial 5′-nucleotidases from seawater and freshwater organisms participate in the regeneration of phosphate from dissolved organic phosphorus compounds and provide phosphates for phytoplankton. Thus, these enzymes have an ecological function in the recycling of nutrients in aqueous habitats (Zimmermann 1992).

The specific substrate specificities and periplasmic or extracellular localizations of bacterial 5′-nucleotidases allow these enzymes (in combination with nucleases) to provide cells with phosphate, carbon, and nitrogen through DNA degradation. This is a useful strategy to survive under conditions of phosphate limitation and when extracellular DNA is available. Indeed, the key role of UshA in the growth of bacteria on nucleotides, RNA, and DNA as a source of carbon and phosphorus has been demonstrated in *Shewanella* spp. (Pinchuk et al. 2008), *E. coli* (Kakehi et al. 2007), *V*. *cholerae* (McDonough et al. 2016), *C. glutamicum* (Rittmann et al. 2005), and *B. subtilis* (Chambert et al. 2003).

The periplasmic location of protein homologs of *E. coli* UshA and their very high catalytic efficiencies (UshA *k*_cat_/*K*_M_ values that are three orders of magnitude higher than the average value (10^5^ s^–1^ M^–1^) for more than 5000 enzymes presented in the BRENDA database (Bar-Even et al. 2011)) make them a powerful enzymatic tool for the assimilation of exogenous nucleotides, CDP-alcohols, UDP-sugars, DNA, and other phosphorylated compounds, which cannot be transported through the cell membrane. Two *E. coli* enzymes with 5′-nucleotidase activity secreted into the periplasm, UshA and AphA, are essential for growth on purine nucleotides as the sole carbon source (Kakehi et al. 2007). Along with AphA, other class B acid phosphatases play essential roles in the generation, acquisition, and mobilization of inorganic phosphate and critical roles in phosphoryl relay systems involved in signal transduction pathways (Rossolini et al. 1998). The extracellular production of *C. glutamicum* UshA and *B. subtilis* YfkN under phosphate starvation conditions was enhanced, suggesting that they play a role in phosphate metabolism.

However, the functions of periplasmic 5′-nucleotidases may be broader than just the assimilation of exogenous phosphorylated compounds as nutrients. Thus, the periplasmic acid phosphatase AphA from *E. coli* plays an as of yet poorly investigated role in regulating DNA replication (Reshetnyak et al. 1999)*.* AphA was shown to bind hemimethylated (but not fully methylated) DNA at the replication origin (*oriC*) and is probably involved (along with the SeqA protein) in *oriC* sequestration at the membrane, preventing the reinitiation of chromosome replication from newly formed *oriC.*

Moreover, periplasmic 5′-nucleotidases can influence the virulence properties of pathogens, increasing the damage caused by their invasion. The inhibitory effect of *E. coli* UshA activity, yielding adenosine overproduction, on the activity of the human tyrosine kinase p56^lck^ and other protein kinases indicates the potential role of periplasmic 5′-nucleotidases of enteropathogenic *E. coli* and other pathogenic bacteria in subverting host defense (Berger et al. 1996). Periplasmic acid phosphatases from *Pseudomonas aeruginosa* and other virulence factors are released into host cells, where they degrade cellular components, thereby aiding infection by the pathogen (Kadurugamuwa and Beveridge 1997). Alves-Pereira et al. (2008) suggested that in the case of intracellular infection, the CDP-alcohol hydrolase activity of UshA from pathogenic *Yersinia* species can disrupt CDP-ethanolamine and CDP-choline, key intermediates in the host’s phospholipid biosynthetic pathways, thus affecting the synthesis of cell wall components. Together with extracellular nucleases and other phosphorylases, the *V. cholerae* 5′-nucleotidase UshA can destroy neutrophil-secreted DNA (as part of NETs), thus affecting the host’s defense systems and protecting *V. cholerae* from NET attack (McDonough et al. 2016).

The fact that class B acid phosphatases secreted into the periplasm have been found mainly in pathogenic bacteria capable of causing a wide range of nosocomial infections suggests that these enzymes, in addition to the assimilation of phosphomonoesters, play a role in the pathogenicity of these microbes. Extracellular, secreted ATP-hydrolyzing enzymes from *B. cepacia* (Melnikov et al. 2000) and *P. aeruginosa* (Zaborina et al. 1999) have been shown to modulate the external ATP levels of macrophages and mast cells, leading to their accelerated death (presumably through the activation of purinergic nucleotide receptors), and thus play an important role in evading host defense.

UshA from *S. oneidensis*, which can respire extracellular substrates through extracellular electron transport, has been shown to play an additional important physiological role. Along with direct electron transfer to substrates (such as elemental sulfur and oxidized metals) through a number of cytochromes, the bacterium uses extracellular flavin molecules to shuttle electrons to the terminal electron acceptor (von Canstein et al. 2008). *S. oneidensis* UshA is involved in the processing of extracellular flavin electron shuttles (Covington et al. 2010). FAD, which cannot be transported in the extracellular space, is transported by the Bfe exporter into the periplasm, where it is hydrolyzed by UshA. The product of FAD hydrolysis, FMN, diffuses through outer membrane porins and accelerates extracellular electron transfer.

### Intracellular 5′-nucleotidases

As described above, periplasmic, surface-located, and extracellular 5′-nucleotidases are involved in the regulation of extracellular nucleotides, are responsible for the assimilation of extracellular nucleotides as nutrient sources, and contribute to immune evasion. Intracellular nucleotidases are involved in regulating intracellular nucleotide pools for the maintenance of correct DNA and RNA synthesis. Many soluble, intracellular phosphatases with 5′-nucleotidase activity have been found in gram-positive and gram-negative bacteria and biochemically characterized, but the definitive biological roles of only a few of these enzymes have been experimentally confirmed. Among these is the *E. coli* UMP phosphatase UmpH, which was found to degrade “overflow” UMP nucleotides and is required for optimal growth in response to environmental pyrimidine intermediates (Reaves et al. 2013). With a significantly higher Michaelis constant (*K*_*m*_ of 0.12 mM) than the normal steady-state UMP concentration (0.052 mM), UmpH converts UMP to uridine only under conditions of UMP overproduction, thus decreasing intracellular UMP concentrations even in the presence of deregulated pyrimidine biosynthetic flux. A similar function in pyrimidine homeostasis was shown for the SurE family 5′-nucleotidase UmpG (Reaves et al. 2013). *E. coli* SurE is involved in the regulation of dNTP and NTP pools, plays a significant physiological role in the stress response, and is required for the survival of cells in the stationary growth phase (Li et al. 1994). The HDDSF 5′-nucleotidase YfbR plays an important role in the deoxycytidine pathway and de novo synthesis of thymidylate in *E. coli*, catalyzing the hydrolysis of 2′-deoxycytidine-5′-monophosphate to deoxycytidine (Weiss 2007).

Moreover, several HADSF phosphatases were shown to directly participate in the histidine, serine, and FMN (RF) biosynthesis pathways (Rangarajan et al. 2006; Kuznetsova et al. 2006; Haase et al. 2013). The *B. subtilis* HADSF phosphatases YcsE, YwtE, and YitU, which exhibit various affinities for ARPP and FMN, are involved in the synthesis and fine-tuning of intracellular pools of important flavins, such as RF, FMN, and FAD (Sarge et al. 2015; Yusupova et al. 2020). These data suggest the wider role of intracellular, microbial phosphatases/5′-nucleotidases in the HADSF in the regulation of cellular metabolism, which is not limited by the control of intracellular nucleotide pools but also extends to the control of pools of other key cellular metabolites.

Moreover, 5′-nucleotidases are used to protect cells from the adverse effects of nonendogenous metabolites. For example, YjjG functions as a housekeeping phosphatase in vivo (Titz et al. 2007). This nucleotide phosphatase can dephosphorylate a wide range of noncanonical pyrimidine derivatives (5-fluoro-2′-deoxyuridine, 5-fluorouridine, 5-fluoroorotic acid, 5-fluorouracil, etc.), thus preventing the incorporation of these potentially mutagenic compounds into DNA and RNA.

Interestingly, insertional disruption of the *nagD* gene (orthologous to *E. coli umpH*) in the *S. aureus* genome reduced the virulence of this bacterium (Begun et al. 2005), supporting the idea that intracellular 5′-nucleotidases, similar to surface-located and periplasmic enzymes, may contribute to the virulence of pathogenic strains. *E. coli* UmpH orthologs are also present in other human pathogens such as *S. aureus* (Tremblay et al. 2006).

The catalytic promiscuity and substrate ambiguity of HADSF phosphatases/5′-nucleotidases are key factors for the evolution of enzymes and acquisition of new biological functions. While the core Rossmann-like folds of these types of enzymes are rather conserved, the inserted cap domain presents new surfaces for substrate interactions, adding a sophisticated means of substrate recognition, diversifying enzyme function, and bestowing new advantageous properties (Pandya et al. 2014).

## Regulation of microbial 5′-nucleotidase activity

Since alterations in the nucleoside system play a very important role in modulating both physiological and pathophysiological processes in humans, the modulation of 5′-nucleotidase activity may be a promising therapeutic tool for treating various diseases (Kovács et al., 2013); therefore, the regulation of mammalian 5′-nucleotidase activity has been investigated in-depth (Pesi et al. 1994; Camici et al. 2020). Much less is known about the regulation of microbial 5′-nucleotidase activity. Nucleotides are components of several vital cellular processes. 5′-Nucleotidases, enzymes involved in nucleotide metabolism, are tightly regulated to ensure homeostasis, preventing both wasteful catalytic cycles and the toxicity of “overflow” metabolism. Similar to the activity of other enzymes, 5′-nucleotidase activity can be controlled through regulating expression of the respective gene and through posttranslational regulations, such as allosteric regulation.

### Expression regulation

Phosphate limitation induces genes that encode phosphate-liberating enzymes to provide sufficient inorganic phosphate for survival under conditions of phosphate starvation. The *E. coli* alkaline phosphatase gene *phoA* is part of the phosphate regulon, and its expression is positively regulated by the transcriptional regulator PhoB (Makino et al. 1986). The amount of the periplasmic alkaline phosphatase PhoA was significantly increased when *E. coli* cells were starved for phosphate (the conditions of the most common *E. coli* environment, the human gut) and reached approximately 6% of the total protein (Torriani 1960; Garen and Levinthal 1960). Similarly, the 5′-nucleotidase genes *ushA* from *C. glutamicum* and *yfkN* from *B. subtilis*, which are part of phosphate starvation regulons, exhibit increased expression under conditions of phosphate deprivation (Allenby et al. 2005; Rittmann et al. 2005; Kocan et al. 2006). However, despite the similar roles of numerous UshA orthologs in phosphate assimilation, phosphate-dependent regulation of the expression of the corresponding genes is not universal. Thus, expression of the *E. coli* and *V. cholerae ushA* genes does not depend on phosphate availability (Burns and Beacham 1986; McDonough et al. 2016). At the same time, the possible dependence of *E. coli ushA* transcription on the growth phase was suggested based on the presence of three *ushA* transcripts and changes in the relative proportions of these transcripts during growth-phase regulation (Burns and Beacham 1986). Nucleotide-induced expression of a membrane-bound 5′-nucleotidase from *V. parahaemolyticus*, which utilizes ATP, ADP, or AMP as a sole carbon source, was suggested based on the approximately three times higher enzyme activity in cells grown in the presence of these nucleotides compared to that in cells grown in nucleotide-free medium (Sakai et al. 1987). The presence of antibodies against a cell wall–anchored 5′-nucleotidase from *S*. *pyogenes* in serum samples from patients indicates that the enzyme is produced under infectious conditions (Reid et al. 2002).

Few published studies on the regulation of bacterial intracellular 5′-nucleotidase gene expression have demonstrated their increased expression upon partial nucleotide limitation (Dhariwal et al. 1982); under oxidative, salt, and thermal stresses; and in the presence of various toxic compounds, such as diamide and ethanol (Terakawa et al. 2016). Moreover, positive autoregulation of the expression of *B. subtilis yutF*, which encodes a HADSF phosphatase/5′-nucleotidase, was revealed (Zakataeva et al. 2016).

### Posttranslational regulation

Most enzymes are directly controlled by alteration of their catalytic activity by conformational changes in the protein in response to phosphorylation and the binding of small molecules or proteins.

HADSF phosphatases and the response regulator receptor domains in two-component signal transduction systems have very similar active sites and catalyze the same fundamental chemistry (Ridder and Dijkstra 1999; Immormino et al. 2015). Signal transduction systems allow organisms to sense and respond to changes in various environmental conditions (Stock et al. 2000). These systems normally consist of a membrane-bound histidine kinase, which detects a signal, and a response regulator, which, in its phosphorylated form, executes a cellular response (Gao et al. 2007). Interestingly, some response regulators consist of an isolated receptor domain (lacking an effector domain) and can regulate target effectors through their own phosphorylation by small phosphodonors (Lukat et al. 1992). It can be assumed that some HADSF phosphatases act in a similar way, interacting with a substrate as phosphodonor to form an intermediate phosphorylated form that is capable of activating a specific cellular response (Zakataeva et al. 2016).

In some cases, the activity of the enzyme changes in response to changes in the conformation of the protein and the interaction between subunits due to the binding of a regulatory molecule. This type of regulation is often responsible for controlling metabolic pathways through feedback inhibition. Many examples of feedback inhibition of an upstream anabolic enzyme by a downstream metabolite have been described (Sander et al. 2019). However, feedback activation, in which a subsequent metabolite activates an upstream catabolic enzyme, is less common. The cytosolic 5′-nucleotidase from *Legionella pneumophila*, LpcN-II, was shown to activate GTP feedback to deplete the precursor (GMP) and maintain control of its concentration. The multiple regulation of these enzymes and structural reorganization of the enzyme subunits upon binding of the activator have been suggested as likely mechanisms of homotropic and heterotropic activation by the substrate and activator, respectively (Srinivasan et al. 2014).

In many cases, protein activity is regulated by protein–protein interactions. Glaser et al. (1967) showed that both the UDP-sugar hydrolase and nucleotidase activities of *E. coli* UshA are inhibited by specific proteins in the cytoplasm. This inhibition may serve to protect cytoplasmic nucleotides from degradation prior to the secretion of UshA to the periplasm (Innes et al. 2001). Many 5′-nucleotidases are composed of several subunits, and interactions between polypeptide chains are important for the regulation of protein activity. Thus, activity regulation of some 5′-nucleotidases can be achieved through the regulatory influence of their own accessory domains, which may play an inhibitory or activating role. For example, *E. coli* UshA consists of an N-terminal catalytic domain and an accessory substrate-binding C-terminal domain, which regulates substrate utilization. The catalytic function of the enzyme depends on a 96° rotation between the C-terminal and N-terminal domains. The N-terminal domain alone was able to hydrolyze in vitro substrates such as ATP, ADP, and pNPP (albeit with lower affinities than the full-length enzyme) but could not hydrolyze AMP (Krug et al. 2013).

An interesting mechanism for the activity regulation of metallophosphatase superfamily 5′-nucleotidases, which use a specific metal ion to activate a water molecule as an initial phosphate acceptor, was suggested. Since the efficiency of the hydrolysis of various substrates by these enzymes might depend on the type of metal ion used, enzyme activity and even affinity for a certain substrate might be regulated by local metal concentrations. Thus, the chelation of different groups of metals by intracellular and secreted 5′-nucleotidases provides an additional level of regulation of the activity of these enzymes and may also influence the substrates that they utilize in these different compartments (Valdez et al. 2014; Matange et al. 2015).

## Approaches to find new 5′-nucleotidases

Typically, functional annotation methods are based on database searches for proteins homologous to experimentally characterized proteins using alignment search tools such as BLAST (Altschul et al. 1990, 2005). If the sequence similarity is high enough, proteins can be designated “hypothetical” or “putative,” and their specificity and function should be verified experimentally. However, if the amino acid sequence similarity is low, for example, due to a large phylogenetic distance between organisms, the probability of finding isofunctional orthologs in another organism is significantly reduced. In such cases, an iterative search for a position-specific score matrix using PSI-BLAST (Altschul et al. 1997) might be helpful. However, in some cases, to identify distant relatives of a protein family, the respective encoding genes should be directly selected based on the specific enzymatic reactions of the gene products. According to the published data, the identification of new genes that encode enzymes with 5′-nucleotidase activity in microbial genomes and the functional assignment of their products have been performed by using all of the abovementioned approaches. 

In *E. coli* and *B. subtilis*, intracellular HADSF phosphatases/5′-nucleotidases have mainly been found and functionally characterized by screening putative phosphatases with a set of phosphorylated compounds, including various nucleotides (Kuznetsova et al. 2006; Proudfoot et al. 2004; Huang et al. 2015; Terakawa et al. 2016; Zakataeva et al. 2016). 

An alternative method to search for new *B. subtilis* genes that encode enzymes with 5′-nucleotidase activity is based on “shotgun” cloning followed by the direct selection of recombinant plasmids containing DNA fragments with 5′-nucleotidase-encoding genes (Yusupova et al., 2020). Such selection was performed by the growth of a specially constructed host, *E. coli* GS72 (TG1 deoD gsk-3), at high inosine and guanosine concentrations. Contrary to the wild-type strain, this *deoD*/*gsk* mutant is sensitive to the purine nucleosides inosine and guanosine due to the inhibitory effect of their respective phosphorylated derivatives on 5-phosphoribosyl-1-pyrophosphate (PRPP) synthetase activity. The overexpression of genes involved in purine mononucleotide hydrolysis abolished the growth arrest of *E. coli* GS72 cells in the presence of purine nucleosides. 

The genes that encode the cell wall–anchored 5′-nucleotidase (adenosine synthase) AdsA in *S. aureus* and *B. anthracis* were also identified using a phenotypic selection approach based on the inability of the *adsA* insertion mutants to escape host immune responses (Thammavongsa et al. 2009).

## Practical applications of microbial 5′-nucleotidases

5′-Nucleotidases/phosphatases are involved in many fundamental biochemical processes and play a key role in the phosphorus cycle. The practical applications of nonspecific phosphatases are well known. Thus, alkaline phosphatases, nonspecific phosphomonoesterases that can cleave phosphate ester bonds and catalyze the transfer of a phosphate group, are widely used in molecular biology, clinical research, and environmental toxicity tests (Van Dyk and Pletschke 2011). Since alkaline phosphatases can hydrolyze the phosphate bond in organophosphorus compounds, these enzymes have potential industrial applications in bioremediation for detoxifying residual organophosphorus compounds in the environment.

Another area of application for 5′-nucleotidases/phosphatases is the biotechnological production of nucleotides and nucleosides. The sodium salts IMP and GMP are widely used as *umami* flavor enhancers and formulated in dietary supplements in combination with monosodium glutamate (MSG) to synergistically enhance flavor. In addition, IMP, GMP and their corresponding nucleosides, inosine and guanosine, have a number of beneficial effects related to their antioxidant, neuroprotective, cardiotonic, and immunomodulatory properties (Ledesma-Amaro et al. 2013). Inosine and guanosine are produced from cell cultures by the microbial fermentation of designed strains. To obtain their monophosphate derivatives, IMP and GMP, nucleosides undergo phosphorylation by chemical or enzymatic treatment (Ledesma-Amaro et al. 2013). To provide an efficient enzymatic phosphorylation, mutant variant of acid phosphatase, PhoC from *Morganella morganii*, was applied (Mihara et al. 2000). Some bacterial strains that can excrete purine nucleotides produce IMP and GMP directly by microbial fermentation (Kuninaka 2001). The identification of genes that encode IMP 5′-nucleotidases and their overexpression are a promising approach to construct inosine-producing strains based on *B. subtilis* (Matsui et al. 1982; Asahara et al. 2010). In contrast, specific impairment of IMP and GMP 5′-nucleotidase activity in IMP- and GMP-producing cells is an approach used to increase the efficiency of industrial strains (Furuya et al. 1968).

Modulation of the activity of the membrane-bound, surface-located metallophosphoesterase/5′-nucleotidase UshA from *Shewanella* spp. to increase the secreted concentration of flavin, which is involved in extracellular electron transfer, in addition to other approaches might be useful for increasing the wide range of innovative biotechnological processes that utilize *Shewanella* cells, including environmental bioremediation, microbial electrosynthesis, and energy production in microbial fuel cells (Zou et al. 2018).

Tuning of the expression of genes that encode 5′-nucleotidases, which participate in the histidine, serine, FMN, and RF biosynthesis pathways, can be an important factor to enhance the production of biotechnology-relevant compounds such as amino acids and flavins. For example, the enhanced expression of the 5′-nucleotidase gene *yitU* in *B. subtilis* strains was shown to increase the production of RF and 5-aminoimidazole-4-carboxamide-1-β-D-ribofuranosyl 5′-monophosphate (AICAR) (Yusupova et al. 2020), which are widely used in food technology and the pharmaceutical industry (Schwechheimer et al. 2016; Lobanov et al. 2011).

Another potentially promising area to which 5′-nucleotidases could be applied is the vaccine industry. The problems of antimicrobial resistance and associated morbidity and mortality due to antibiotic-resistant microbial pathogens have become more acute over time. Vaccines act as a tool to prevent life-threatening diseases caused by pathogenic bacteria, to reduce the use of antibiotics, and to alleviate the problem of antimicrobial resistance. Cell wall–anchored 5′-nucleotidases from gram-positive cocci, many of which are the most significant human bacterial pathogens, have characteristics that make them promising candidates for vaccine development (Soh et al. 2020). These characteristics are the high level of sequence conservation among 5′-nucleotidase-encoding genes, the accessibility of the enzymes at the cell surface, the presence of these pathogens in a wide variety of clinical isolates, and their important role as virulence factors in immune evasions. Thus, enzymatically inactive mutants of class C acid phosphatase *e* (P4) were shown to be potential *H. influenzae* vaccine candidates (Green et al. 2005). Furthermore, the potential to apply the *e* (P4) orthologous protein from *C. perfringens*, the acid phosphatase CppA, as a diagnostic tool for the differentiation of clostridial species and as a candidate for vaccine development against these bacteria was suggested (Reilly et al. 2009). Recently, the cell wall–anchored 5′-nucleotidase (adenosine synthase) AdsA from *S. aureus* was included in a multivalent vaccine formulation (Deng et al. 2019). Moreover, a live vaccine for the encapsulated zoonotic pathogen *Streptococcus suis* was developed by multigene deletion of five virulence factors, including the 5′-nucleotidase gene ssads (Li et al. 2019).

## Conclusions and future prospects

5′-Nucleotidases encompass a huge variety of enzymes and are found in all domains of life, including all bacterial phyla; furthermore, organisms encode between a few and several dozen of the respective genes. These enzymes play very important and multifaceted roles in cells. 5′-Nucleotidases differ in molecular structure, hydrolytic mechanism, and cellular localization. In many cases, these enzymes can cleave phosphorus from not only mononucleotide phosphate molecules but also a number of other phosphorylated compounds, both intracellular metabolites and those present in the surrounding space. Generally, they have broad substrate specificity but show a preference for certain substrates. An interesting feature of 5′-nucleotidases is that they do not adhere to the classical “one enzyme for one substrate” model; instead, these enzymes use a “one substrate to many enzymes” model. This feature may serve to provide sufficient turnover (without optimizing the efficiency of a single specific enzyme) to metabolize large pools of similar substrates. Overlap between substrate spectra may also promote the development of new metabolic functions in response to environmental requirements. The current understanding of the roles and functions of microbial 5′-nucleotidases still lags behind that of mammalian 5′-nucleotidases. The key functions and main substrates for most microbial 5′-nucleotidases have not yet been found despite the wide screening of various phosphorylated compounds as their possible substrates. Studying the evolution of these promiscuous enzymes may be helpful for the development of new approaches in protein engineering and synthetic biology.
